# Implementation of environmental enrichment after middle age promotes healthy aging

**DOI:** 10.18632/aging.101502

**Published:** 2018-07-20

**Authors:** Travis McMurphy, Wei Huang, Nicholas J. Queen, Seemaab Ali, Kyle J. Widstrom, Xianglan Liu, Run Xiao, Jason J. Siu, Lei Cao

**Affiliations:** 1 Department of Cancer Biology and Genetics, College of Medicine, The Ohio State University, Columbus, OH 43210, USA; * Equal contribution

**Keywords:** environmental enrichment, aging, glucose tolerance, BDNF, steatosis, adipose tissue

## Abstract

With increases in life expectancy, it is vital to understand the dynamics of aging, their interaction with lifestyle factors, and the connections to age-related disease processes. Our work on environmental enrichment (EE), a housing environment boosting mental health, has revealed a novel anticancer and anti-obesity phenotype mediated by a brain-fat axis: the hypothalamic-sympathoneural-adipocyte (HSA) axis in young animals. Here we investigated EE effects on healthspan and lifespan when initiated after middle age. Short-term EE for six weeks activated the HSA axis in 10-month-old mice. Long-term EE for twelve months reduced adiposity, improved glucose tolerance, decreased leptin levels, enhanced motor abilities, and inhibited anxiety. In addition to adipose remodeling, EE decreased age-related liver steatosis, reduced hepatic glucose production, and increased glucose uptake by liver and adipose tissue contributing to the improved glycemic control. The EE-induced liver modulation was associated with a suppression of protein kinase Cε. Moreover, EE down-regulated the expression of inflammatory genes in the brain, adipose, and liver. EE initiated at 18-month of age significantly improved glycemic control and showed a trend of positive impact on mean lifespan. These data suggest that EE induces metabolic and behavioral adaptations that are shared by factors known to increase healthspan and lifespan.

## Introduction

The incidence of age-related diseases such as cancer, cardiovascular disorders and neurodegenerative diseases rises with an increase in life expectancy. Therefore, it is vital to understand more about the dynamics of aging, particularly how they interact with various environmental and lifestyle factors, and the connections between disease processes and aging in order to develop more effective strategies to prevent, diagnose and treat age-related diseases. Numerous studies on model organisms have revealed an interaction between genes and environment in determining healthy lifespan, i.e., the age free of significant diseases [1,2]. Caloric restriction (CR) without malnutrition (often reduced calorie intake by 40% compared to *ad libitum*) is, by far, the most robust and reproducible approach to delay the onset of age-related disorders and extend lifespan in a wide range of model organisms from yeast to monkeys [1]. Intermittent fasting (IF; a diet with reduced meal frequency, for example, alternative-day feeding) also induces resistance to toxicity and stress, and extends lifespan [3]. The metabolic and physiological characteristics of CR that may contribute to its anti-aging capacity include reduced adiposity, higher insulin sensitivity, improved lipid profiles, reduced oxidative stress and inflammation [4–8]. Over the past few years, we have used a eustress model— environmental enrichment (EE)— that has profound impacts on brain structure and function [9], to study how physical and social environments modulate physiology and disease risk and progression. We demonstrate that EE leads to leanness, resistance to diet-induced obesity (DIO), and inhibition of melanoma and colon cancer [10–13]. The anticancer effects of EE have been confirmed and expanded by other researchers to breast cancer [14], pancreatic cancer [15], and glioma [16]. Our mechanistic studies elucidate one key underlying mechanism: the activation of a specific neuroendocrine brain-adipocyte axis, the hypothalamic-sympathoneural-adipocyte (HSA) axis [17]. The physical, social, and cognitive stimuli provided by EE induces brain-derived neurotrophic factor (BDNF) in the hypothalamus and thereby elevates the sympathetic tone preferentially to the adipose tissue. The resulting adipose tissue remodeling, including the white-to-brown phenotypic switch and the suppression of leptin, results in an anti-obesity and anticancer phenotype. Some of the features of EE overlap with CR, and several genetically modified mouse models linking reduced adiposity to longevity, including protein phosphatase and tensin homologue (PTEN) transgenic mice (Pten^tg^) [18], fat-specific insulin receptor knock-out (FIRKO) [19–21], the translational inhibitor 4E-BP1 (Eif4ebp1-/-) knock-out [22], C/EBPß knock-in (ß/ß) [23], and c-Cbl knock-out [24] mice. EE initiated in young male mice led to over 60% reduction of abdominal fat when mice were fed a normal chow diet (NCD), whereas body weight was identical to the mice with standard laboratory environment (SE) [11]. Muscle mass was increased in EE and hypothalamic BDNF overexpressing mice. Both environmental and genetic activation of the HSA axis is particularly efficient in decreasing adiposity, allowing the dissociation of fat loss from weight loss, which is difficult to achieve with other interventions [25]. Furthermore, the HSA axis activation alleviates obesity-associated insulin resistance, alleviates hyperglycemia and dyslipidemia [11,12], alters adipokine levels with higher adiponectin and lowers leptin expression in adipose tissue as well as in circulating blood [10,12], suppresses tumor progression and metastasis [10,26], and enhances immunocompetence [10,26,27], all mimicking CR [1, 5, 28, 29]. To date, the majority of studies on EE and aging examine the effects on cognitive decline and neurodegenerative diseases, showing that EE could reverse age-related neural, cognitive and behavioral impairments [30–33]. However, scarce evidence is available on the effects of EE on peripheral systems and healthspan or lifespan. Here, we investigated the effects of EE on healthy aging and lifespan from a unique perspective of the recently characterized HSA axis.

## RESULTS

### Short-term EE activates the HSA axis in middle age female mice

**Figure 1:**
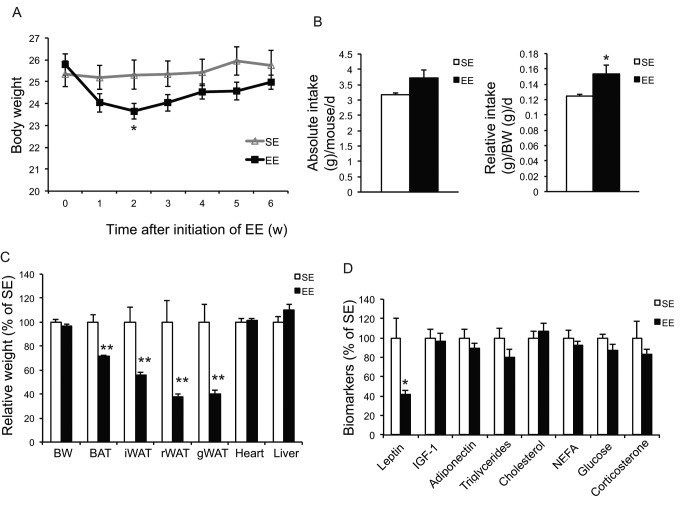
**Short-term EE activates the HSA axis in 10-month old mice.** (**A**) Body weight (n=10 per group). (**B**) Absolute (left) and relative (right) food intake. (**C**) Body and tissue weight at sacrifice after 6-week EE (n=10 per group). (**D**) Serum biomarkers at sacrifice (n=10 per group). * *P*<0.05, ** *P*<0.01. Values are means ± SEM.

**Figure 2:**
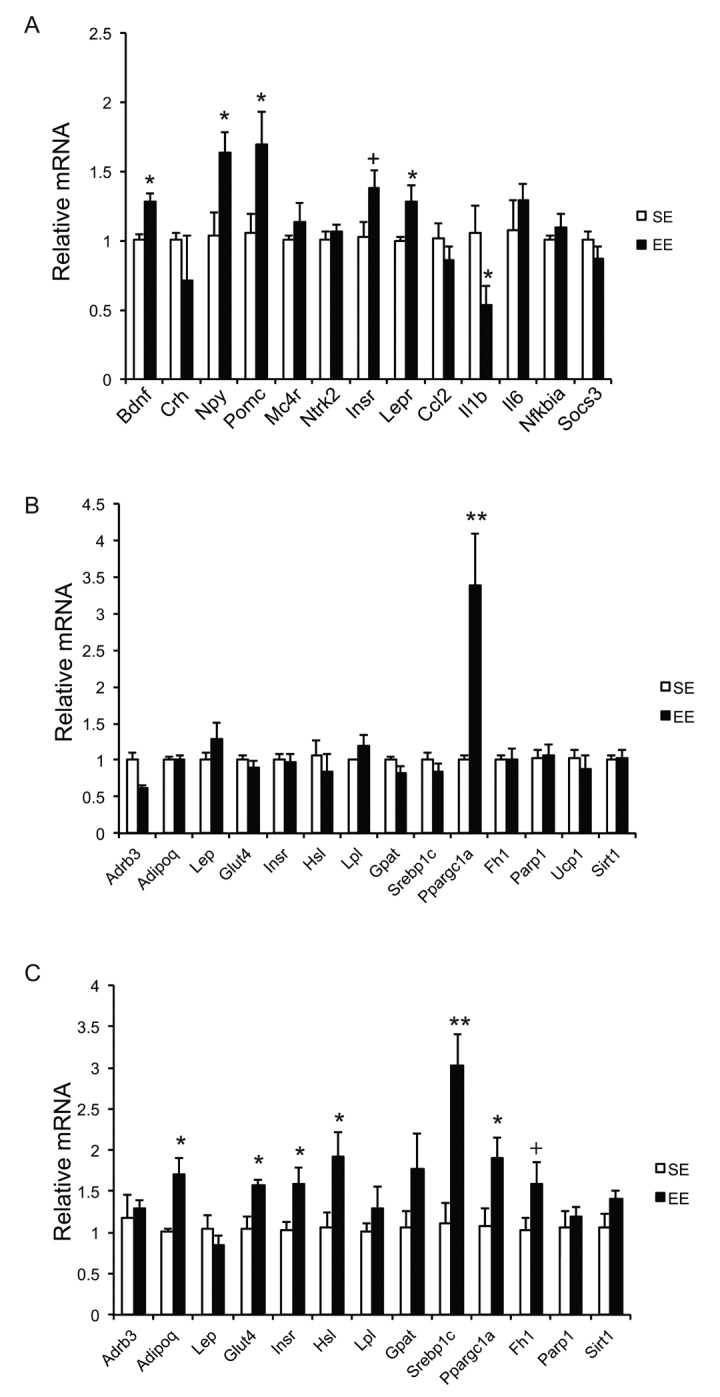
**Gene expression profiles after short-term EE in 10-month old mice.** (**A**) Hypothalamus. (**B**) BAT. (**C**) rWAT. n=5 per group. * *P*<0.05, ** *P*<0.01, + *P*=0.06. Values are means ± SEM.

Our previous studies on EE were performed on young male mice, often initiated immediately after weaning at the age of 3 weeks. To prevent the risk of fighting in group housed older male mice, we used female mice in these aging studies. First, we randomly assigned female mice at the age of 10 months to live in SE or EE for 6 weeks. Body weight and food intake were monitored weekly. EE slightly decreased body weight (Figure 1A), but significantly increased food consumption relative to body weight (Figure 1B). Robust reduction of adiposity was observed in EE mice in both brown adipose tissue (BAT) and three white adipose (WAT) depots: the subcutaneous inguinal WAT (iWAT), abdominal retroperitoneal WAT (rWAT) and gonadal WAT (gWAT) (Figure 1C). Visceral WAT showed the largest reduction: up to approximately 60% consistent with the activation of the HSA axis observed in young male mice (Figure 1C) [11]. Next, the signature of serum biomarkers often associated with EE in young male mice was examined in the middle-aged female mice (Figure 1D). The decrease of IGF-1, the increase of adiponectin and corticosterone found in young male mice were not observed in the middle age female mice (Figure 1D). However, EE resulted in a significant and large drop of leptin by 60%, consistent with the finding in young mice that leptin is the most pronounced change in serum biomarkers responding to EE [10,11]. Real-time quantitative RT-PCR was used to profile the expression of genes involved in energy homeostasis and inflammation. Bdnf was upregulated significantly in EE mice indicating the activation of the HSA axis (Figure 2A). The expressions of BDNF receptor TrkB (Ntrk2) and its upstream melanocortin-4 receptor (Mc4r) were unchanged (Figure 2A). EE upregulated both the orexigenic neuropeptide Y (Npy) and the anorexic proopiomelanocortin (Pomc). Insulin receptor (Insr) and leptin receptor (Lepr) expression were also upregulated by EE (Figure 2A). Among the inflammatory genes profiled, only interleukin-1b (Il1b) was significantly downregulated (Figure 2A). Consistent with results in young mice [11], rWAT showed the most changes in gene expression profile in response to EE while BAT showed very limited changes (Figure 2B, C). Although the effects of EE in middle age female mice did not completely match those in young male mice, the key features of HSA axis activation, namely upregulation of hypothalamic Bdnf, reduction of adiposity and drop of leptin, were confirmed allowing the continued investigation of EE’s impact on healthy aging.

### Long-term EE improves metabolism

**Figure 3:**
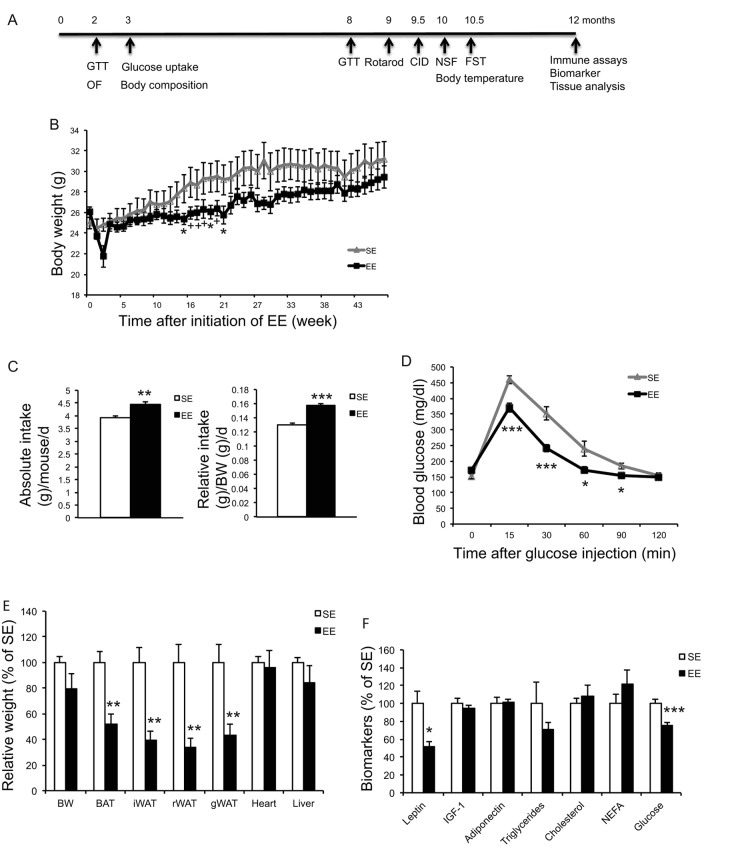
**Long-term EE initiated at middle age reduces adiposity and improves metabolism.** (**A**) Time line. (**B**) Body weight (n=10 per group). (**C**) Absolute (left) and relative (right) food intake 8~10.5 months in EE. (**D**) Glucose tolerance test at 32-week in EE (n=10 per group). (**E**) Body and tissue weight at the age of 22 months after 12-month EE (n=8 per group). (**F**) Serum biomarkers at sacrifice (n=8 per group). * *P*<0.05, ** *P*<0.01, *** *P*<0.001. Values are means ± SEM.

In a long-term EE study, 10-months old female mice were randomly assigned to live in EE or SE for 12 months. Mice were subjected to a series of metabolic measurements and behavior tests as indicated in Figure 3A. Weekly weight monitoring showed that EE reduced weight between 14 to 21 weeks yet there was no difference by the end of the study of 12-months EE (Figure 3B). Food intake was monitored for 10 weeks (8~10.5-month EE) and showed a significant increase (Figure 3C). Rectal temperature was measured after 39-weeks EE and no significant change was observed (SE: 36.8±0.13 °C; EE: 37.2±0.14 °C, *P*=0.10). A glucose tolerance test (GTT) performed at 32-weeks EE showed substantial improvement (Figure 3D). Similar to short-term EE, adiposity was greatly reduced at the end of the 12-months EE study (Figure 3E) and serum leptin level was approximately 50% lower in EE mice (Figure 3F). Furthermore, serum glucose level was also decreased in EE (Figure 3F).

**Figure 4:**
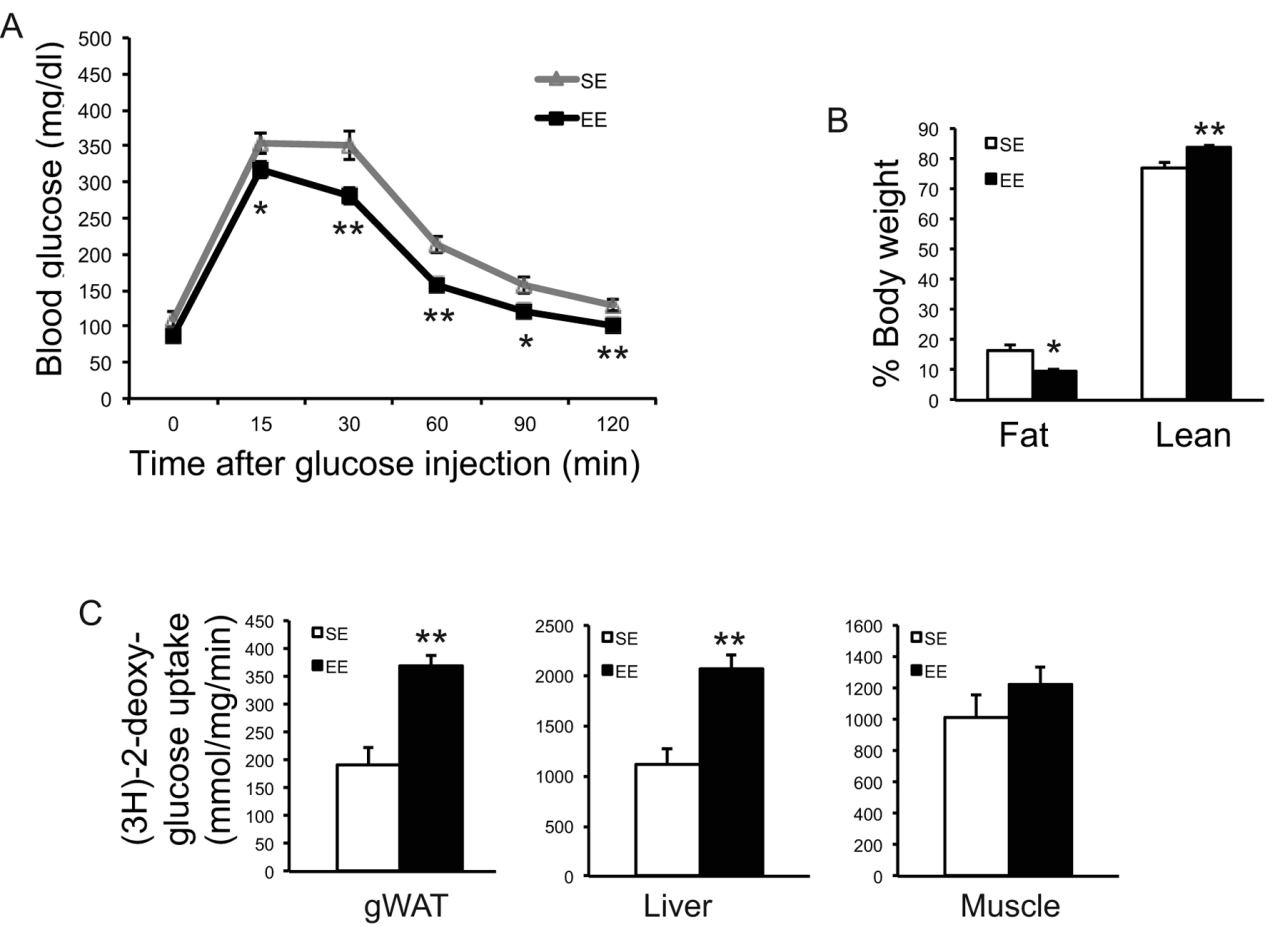
**EE improved glycemic control.** (**A**) Glucose tolerance test of a separate experiment at 8-week in EE (n=10 per group). (**B**) Body composition at 3-month in EE (n=5 per group). (**C**) Glucose uptake assay in gWAT at 12-week in EE (n=5 per group). * *P*<0.05, ** *P*<0.01. Values are means ± SEM.

In a separate cohort of mice, GTT at 2-months EE also showed improved glucose tolerance (Figure 4A). We measured the *in vivo* glucose uptake during a GTT using glucose analog tracer 2-[^3^H] deoxyglucose (2-DG) at 3-months EE. EE significantly increased the glucose uptake by WAT and liver but not skeletal muscle (Figure 4C). EchoMRI was used to measure body composition in this cohort at 3-months EE. Fat mass was reduced by 40% and lean mass was significantly increased with no change of body weight (Figure 4B).

**Figure 5:**
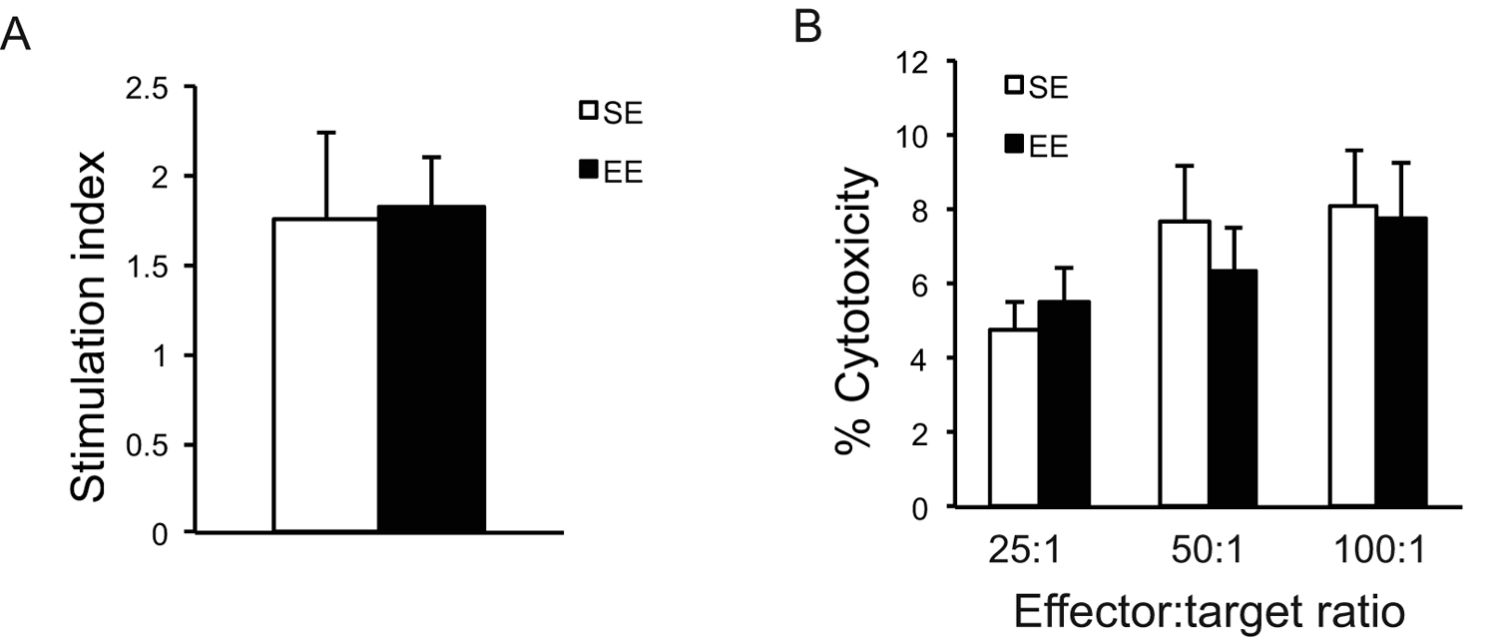
**Immunoassays after long-term EE initiated in 10-month old mice.** (**A**) The proliferative response of splenic lymphocytes to the T cell mitogen Con A. (**B**) NK cell cytotoxicity. n=5 per group. Values are means ± SEM.

In contrast to the strong metabolic effects, long-term EE had no effects on either the proliferative response of splenic lymphocytes to the T cell mitogen Concavalin A (Figure 5A), or the NK cell cytotoxicity against melanoma cells (Figure 5B).

### EE improves motor behavior and reduces anxiety

**Figure 6:**
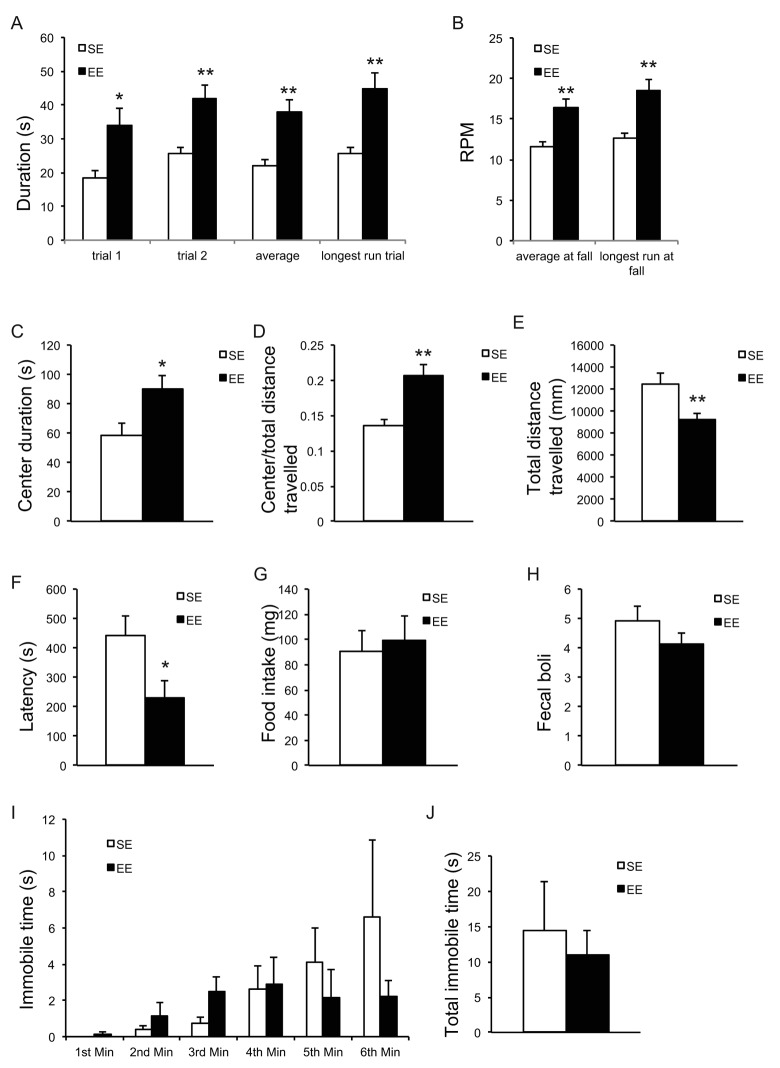
**EE improves motor behavior and reduces anxiety.** (**A**, **B**) Rotarod treadmill test at the age of 19 months after 9-month EE (n=9 per group). Time remaining on rod (**A**), Speed at fall (**B**). (**C**-**E**) Open field test at the age of 12 months after 2-month EE (n=10 per group). Time spent at the center of arena (**C**), ratio of distance travelled in center to total distance (**D**), total distance travelled (**E**). (**F**, **G**) Novelty suppressed feeding test at the age of 20 months after 10-month EE (n=10 SE, n=9 EE). Latency to consumption (**F**), food consumption in standard cage after the test (**G**). (H) Cold induced defecation test at the age of 19.5 months after 9.5-month EE (n=10 SE, n=9 EE). (**I**, **J**) Forced swim test at the age of 20.5 months after 10.5-month EE (n=8 per group). Immobility in each min (**I**), total immobility time (**J**). * *P*<0.05, ** *P*<0.01. Values are means ± SEM.

EE has profound influences on brain structure and function and is often associated with neuroprotection against a variety of toxin- and genetically-induced models of neurological diseases [9]. Previous publications report that EE alleviates motor dysfunction [34–36]. Rotarod treadmill testing measures motor abilities such as balance, coordination, physical condition, and motor-planning [37]. After living in EE for 9 months, mice were subjected to the rotarod treadmill test. Mice in EE were able to remain on the rod for a prolonged period of time (Figure 6A) and for a faster rotating speed before the first fall (Figure 6B) indicating significant improvement in motor abilities. In addition, we performed a battery of anxiety and depression behavior tests. The open field (OF) test is classically used to assess exploratory behavior, general locomotion, and anxiety [38,39]. OF draws on the natural conflict between the tendency to explore a new environment and to avoid an exposed open area [40]. An increase in time spent in the center of the open field is considered to reflect reduced anxiety level. Mice housed in EE for 2 months (a separate cohort of mice) significantly spent more time (Figure 6C) and traveled proportionally more distance (Figure 6D) in the center of the arena, suggesting an anxiolytic effect. Interestingly, EE mice exhibited less locomotion (Figure 6E), which may indicate an enhanced habituation reflecting the more efficient information processing by EE mice— likely a consequence of their greater experience dealing with a new and changing environment [41,42]. The novelty suppressed feeding (NSF) test assesses hyponeophagia, in which exposure to a novel environment suppresses feeding behavior [43]. NSF has been used to study anxiety- and depression-related behaviors, since it is sensitive to anxiolytic and chronic antidepressant treatments. In the NSF assessed after 10-months EE, the latency to eat was significantly reduced in EE mice, suggesting reduced anxiety (Figure 6F). This anxiolytic effect was not due to an enhanced appetitive drive (Figure 6G). In another anxiety behavior test, cold-induced defecation (CID) [44], a trending but not significant decrease in fecal boli was observed in EE mice (Figure 3H). Lastly, the forced swim test is one of the most commonly used rodent behavioral tests for screening antidepressant drugs [45]. No significant effect was observed in FST (Figure 6I, J).

### Long-term EE suppresses inflammatory genes and modulates adipose phenotype

**Figure 7:**
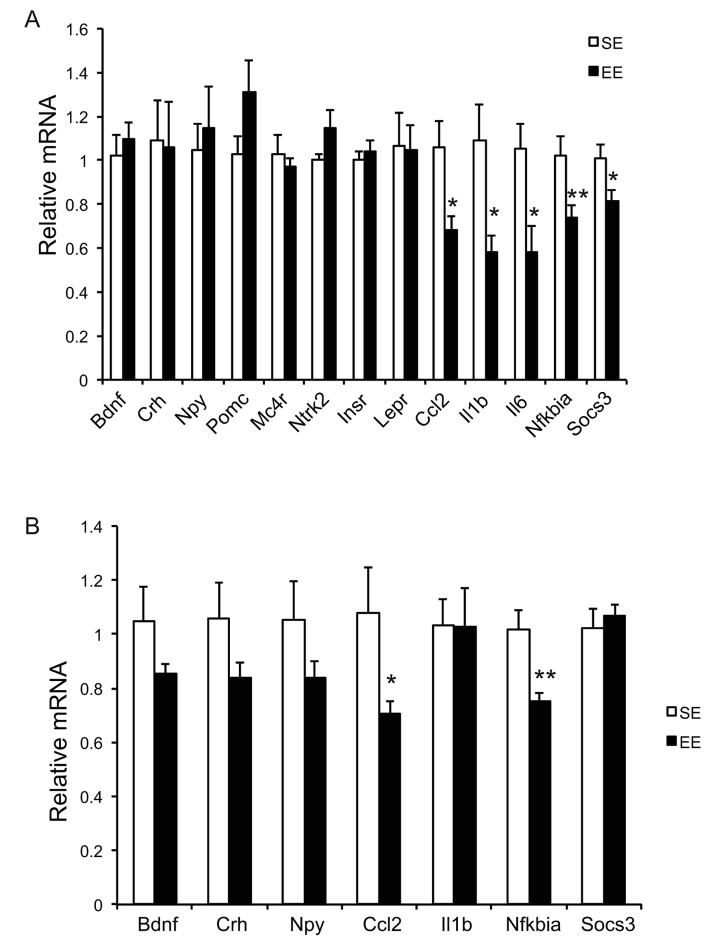
**Brain gene expression profiling at the age of 22 months after 12-month EE.** (**A**) Hypothalamus (n=8 per group), (**B**) Amygdala (n=8 per group). * *P*<0.05, ** *P*<0.01. Values are means ± SEM.

**Figure 8:**
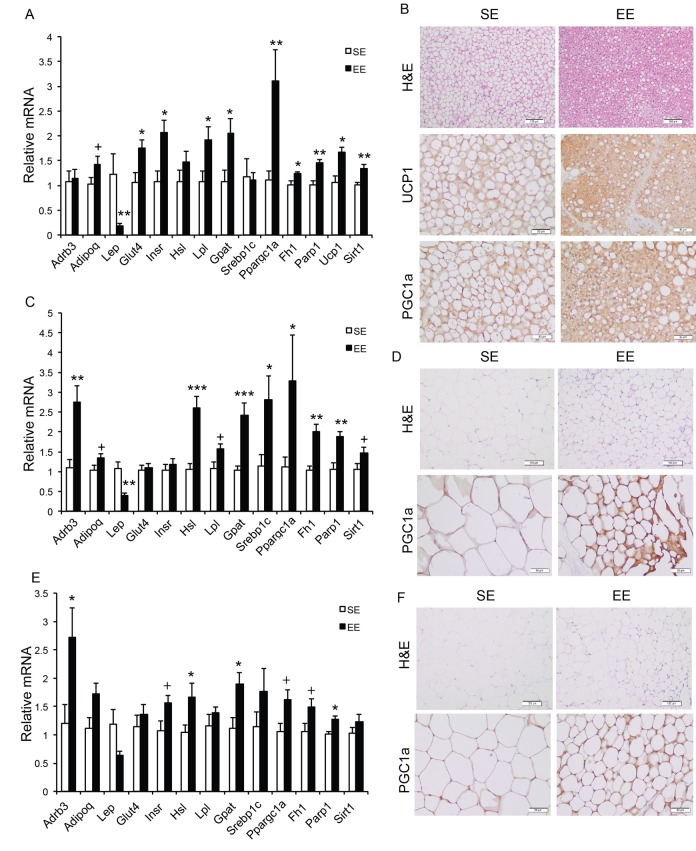
**Analyses of adipose tissues at the age of 22 months after 12-month EE.** Gene expression profiles of (**A**) BAT (n=6 per group), (**C**) rWAT (n=6 per group), and (**E**) gWAT (n=6 per group). (**B**) Immunohistochemistry of BAT. (**D**) Immunohistochemistry of rWAT. (**F**) Immunohistochemistry of gWAT. Scale bar, 100 µm in H&E, 50 µm in UCP1 and PGC-1α staining. * *P*<0.05, ** *P*<0.01, *** *P*<0.001, + *P*=0.06. Values are means ± SEM.

**Figure 9:**
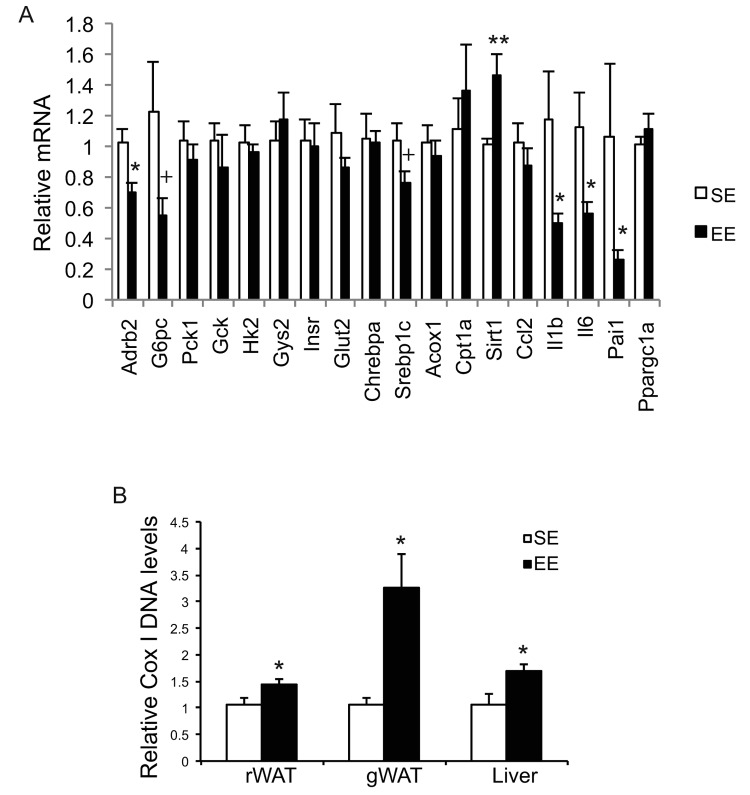
**Analyses of livers at the age of 22 months after 12-month EE.** (**A**) Gene expression profile of liver (n=6 per group). (**B**) Mitochondrial DNA content (rWAT: n=8 per group, gWAT: n=7 per group, liver: n=4 per group). * *P*<0.05, ** *P*<0.01, + *P*=0.06. Values are means ± SEM.

Long-term EE of 12 months led to a pattern of hypothalamic gene expression changes different from that of short-term EE (Figure 7A). The changes in Bdnf and other genes involved in energy homeostasis found in 6-week EE mice (Figure 2A) were not detected. Instead a cluster of genes involved in inflammation including Il1b, Il6, Ccl2 (encoding monocyte chemoattractant protein-1 MCP-1), Nfkbia (encoding nuclear factor of κ light polypeptide gene enhance in B cells inhibitor α), and Socs3 (encoding suppressor cytokine signaling 3) were collectively downregulated in EE mice (Figure 7A). Ccl2 and Nfkbia were also downregulated in the amygdala, a brain area involved in emotionality including anxiety (Figure 7B). Aging is associated with a decline of BAT activity [46]. The BAT of 22-months old mice in SE appeared pale whereas the BAT in EE mice was darker. H&E staining revealed the BAT of EE mice maintained typical BAT morphology of younger mice and was devoid of white adipocyte infiltration often associated with aging (Figure 8B). In contrast to the mild change in gene expression after 6-weeks EE, long-term EE robustly modulated BAT gene expression (Figure 8A). Leptin expression was reduced by over 80% while adiponectin expression showed an increasing trend. Glucose transporter type 4 (Glut4), the major type of glucose transporter in adipose tissue, was significantly induced together with Insr by EE (Figure 8A). Both lipolytic gene Lpl (encoding lipoprotein lipase) and lipogenic gene Gpat (encoding glycerol-3-phosphate acyltransferase) were upregulated. BAT dissipates energy via releasing chemical energy from mitochondria in the form of heat. This process is primarily mediated by uncoupling protein-1 (UCP1) that is a specific BAT marker [47]. Ucp1 was significantly upregulated by EE, suggesting the preservation of proper BAT functions against aging-related loss. The transcriptional coactivator PGC-1α switches cells from energy storage to energy expenditure by inducing mitochondrial biogenesis and genes involved in thermogenesis [48]. Ppargc1a (encoding PGC-1α) was increased over 3-fold in EE BAT (Figure 8A). EE similarly induced Ppargc1a expression in rWAT (Figure 8C) and gWAT (Figure 8E) but not in liver (Figure 9A). Fh1 (encoding mitochondrial fumarate hydratase) and Parp1 (encoding poly ADP-ribose polymerase 1) are associated with CR-induced metabolic adaption [49]. EE induced both Fh1 and Parp1 in BAT and WAT (Figure 8A, C, E). Sirtuins are associated with longevity [50]. EE stimulated Sirt1 expression in BAT, rWAT and liver (Figure 8A, C, Figure 9A). Consistent with the upregulation of mitochondrial genes transcription, mitochondrial DNA contents were increased in adipose tissue and liver (Figure 9B) indicating increased mitochondrial biogenesis. Histology showed that the size of white adipocyte in EE mice was much smaller than that in SE mice (Figure 8B, D, F). Immunohistochemistry demonstrated higher levels of PGC1-α and UCP1 in adipose tissues of EE mice consistent to the gene expression profiling (Figure 8B, D, F).

### EE suppresses liver steatosis and hepatic glucose production

**Figure 10:**
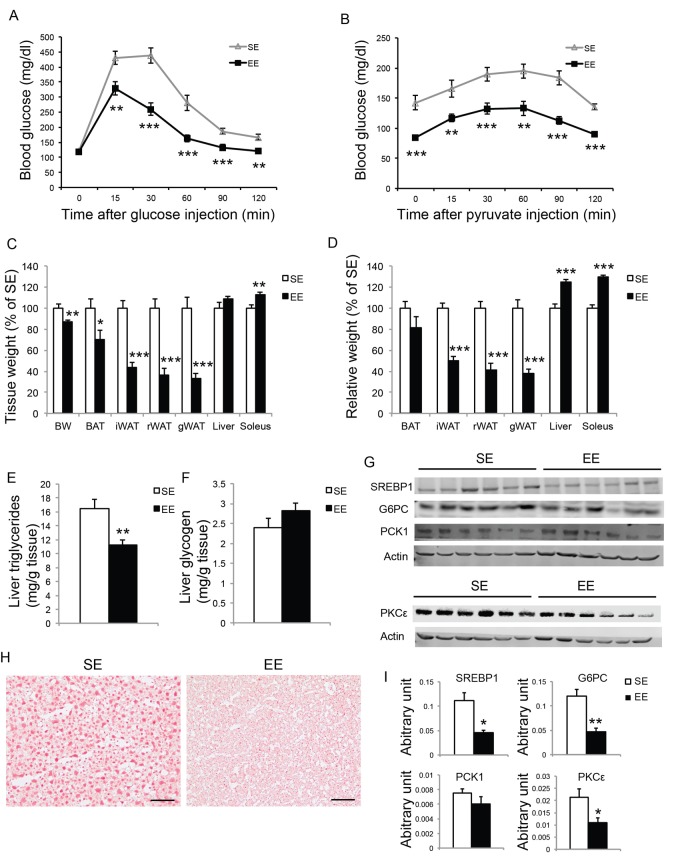
**Short-term EE modulates liver phenotypes in middle age mice.** (**A**) Glucose tolerance test at 6-week in EE (n=10 per group). (**B**) Pyruvate tolerance test at 7-week in EE (n=10 per group). (**C**) Body and tissue weight at sacrifice after 8-week EE (n=10 per group). (**D**) Relative tissue mass calibrated to body weight (n=10 per group). (**E**) Liver triglycerides level (n=10 per group). (**F**) Liver glycogen level (n=10 per group). (**G**) Western blotting of livers. (**H**) Oil red O staining of livers. Scale bar, 50 µm. (**I**) Quantification of western blotting of the livers in (**G**), n=6 per group. * *P*<0.05, ** *P*<0.01, *** *P*<0.001. Values are means ± SEM.

**Figure 11:**
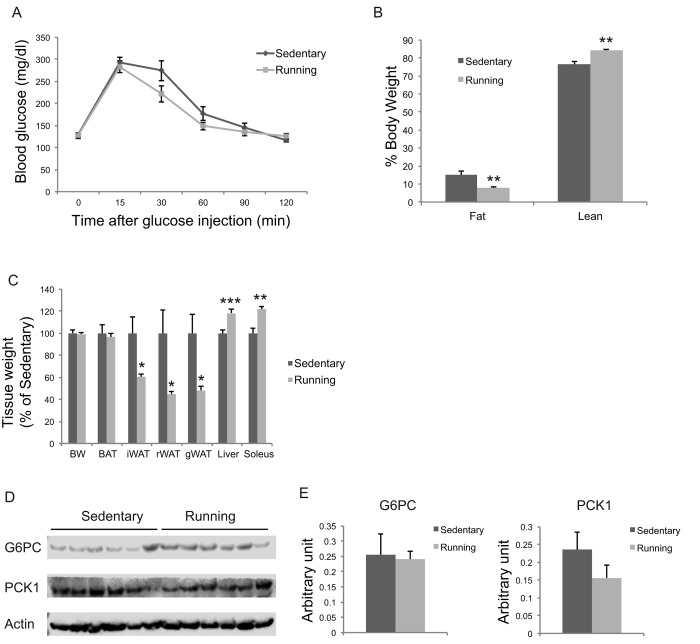
**Metabolic effects of voluntary running in middle-age female mice.** (**A**) Glucose tolerance test after 5-weeks running initiated at 10 months of age. n=11 for Running, n=7 for Sedentary mice. (**B**) Body composition. (**C**) Body and tissue weight at sacrifice after 7-week running. n=12 for Running, n=8 for Sedentary mice. (**D**) Western blotting of livers. (**E**) Quantification of western blotting. n=6 per group. * *P*<0.05, ** *P*<0.01, *** *P*<0.001. Values are means ± SEM.

**Figure 12:**
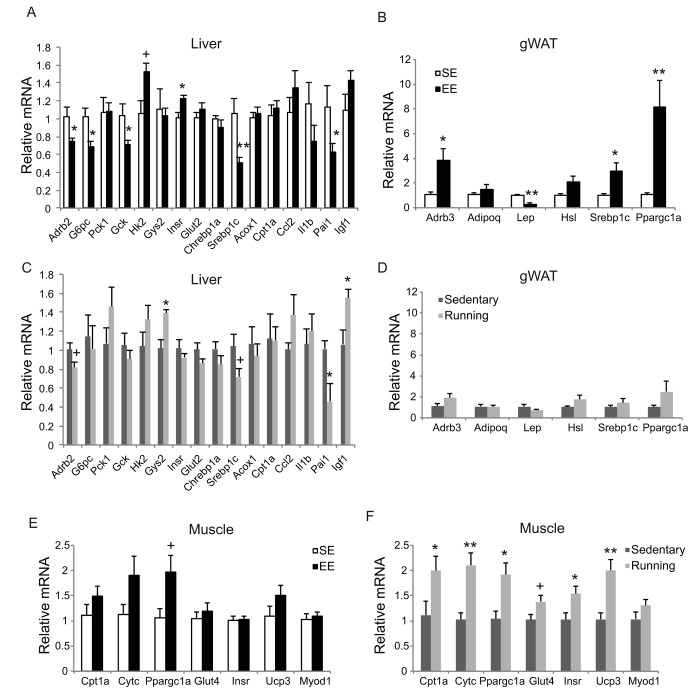
**Gene expression profiles of short-term EE and voluntary running.** Gene expression profiles of liver (**A**), gWAT (**B**), and gastrocnemius muscle (**E**) in EE. n=6 per group. Gene expression profiles of liver (**C**), gWAT (**D**), and gastrocnemius muscle (**F**) in voluntary running. n=5 for sedentary group, n=7 for running group. * *P*<0.05, ** *P*<0.01, + *P*<0.07. Values are means ± SEM.

To further investigate the EE effects on liver in aged mice, we performed another short-term EE experiment. Female mice of 10-month of age were randomized to EE and SE. A GTT at 6-week EE confirmed improved glycemic control (Figure 10A, Figure 11A). To examine hepatic glucose production, a pyruvate tolerance test (PTT) was conducted at 7-week EE after fasting. EE mice displayed significantly lower blood glucose level during PTT (Figure 10B). At the end of this 8-week experiment, EE robustly decreased adiposity and increased lean mass (Figure 10C, D). Non-fast serum glucagon level was not significantly different between the two groups (SE: 20.93±4.33 pg/ml; EE: 35.06±6.99 pg/ml, n=10 per group). Liver glycogen content was not changed by EE (Figure 10F). Aging is associated with hepatosteatosis [51]. EE significantly reduced hepatic triglyceride level compared to SE mice (Figure 10E) while circulating triglyceride level was not significantly different (SE: 58.24±5.82 mg/dl; EE: 46.35±3.32 mg/dl, n=10 per group, P=0.15). Oil Red O staining revealed lipid accumulation in the livers of SE mice at 12 months of age. EE substantially diminished hepatosteatosis (Figure 10H). Western blotting revealed over 50% reduction of SREBP1, the major transcription factor that regulates de novo lipogenesis enzymes, consistent with reduced steatosis [52,53] (Figure 10G, I, Figure 12A). Moreover, the expressions of the two major gluconeogenic enzymes, phosphoenolpyruvate carboxykinase 1 (PCK1) and glucose-6-phosphatase (G6PC), were examined by western blotting and qRT-PCR. G6PC was significantly suppressed at both protein and mRNA levels (Figure 10G, I, Figure 12A). Protein kinase Cε (PKCε) is thought to mediate lipid-induced hepatic insulin resistance and the resulting impaired insulin-induced suppression of hepatic gluconeogenesis [54,55]. EE reduced hepatic PKCε level approximately 50% (Figure 10G, I).

### Physical exercise does not account for EE effects

To investigate the extent to which physical activity accounts for the EE-induced phenotype, we subjected another cohort of 10-month-old mice to voluntary wheel running. Running and sedentary mice showed similar body weight. Similar to EE, running reduced adiposity and increased lean mass (Figure 11B, C). In contrast to EE, running did not significantly affect GTT (Figure 11A) or hepatic G6PC level (Figure 11D, E). Gene expression profiling of the major organs involved in systemic glucose homeostasis (liver, fat, and muscle) revealed distinctive patterns between EE and running (Figure 12). Short-term EE regulated expression of genes involved in gluconeogenesis, glycolysis, lipogenesis, and inflammation (Figure 12A). Running led to fewer changes among this gene expression panel in liver (Figure 12C). Consistent with the HSA axis activation, EE upregulated Adrb3, Srebp1c and Ppargc1a while sharply downregulating leptin in visceral fat (Figure 12B). This adipose gene signature induced by EE was not observed in running mice (Figure 12D). In contrast, EE resulted in minimal changes of gene expression in muscle (Figure 12E) whereas running highly upregulated genes involved in glucose metabolism, fatty acid oxidation, and mitochondrial biogenesis (Figure 12F).

### EE initiated at 18-month of age affects lifespan

**Figure 13:**
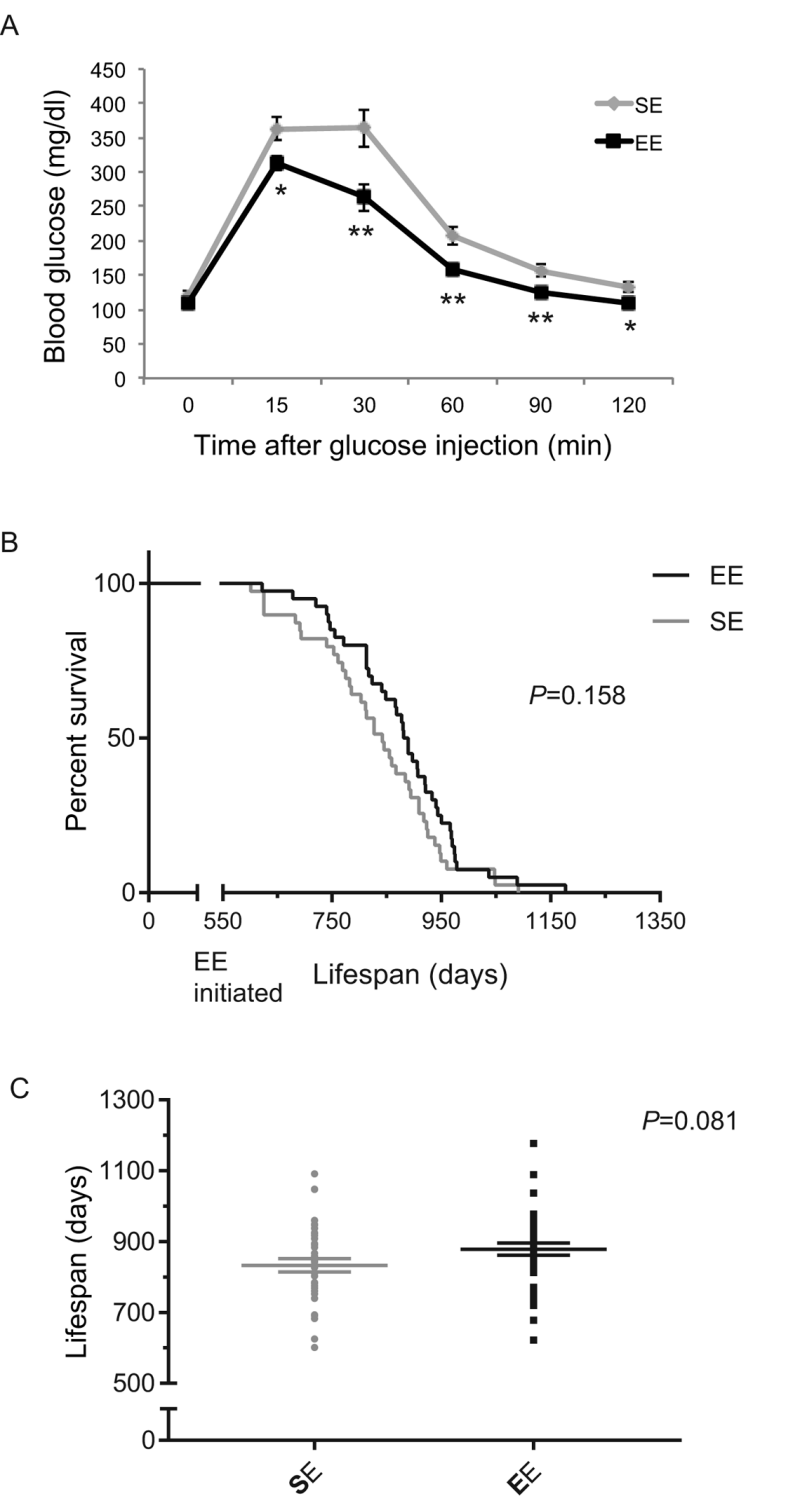
**EE effects on lifespan initiated at 18-month of age.** (**A**) Glucose tolerance test after 3-months EE in one cohort. n=10 per group. * *P*<0.05, ** *P*<0.01. Values are means ± SEM. (**B**) Kaplan-Meier survival curves of combined data of 4 cohorts. n=39 for SE, n=40 for EE. Log-rank test *P* value shown in the figure. (**C**) Means of lifespan. Individual value plot of lifespan. Two-sample T test *P* value shown in the figure.

To investigate whether mice at old age could respond to EE, we initiated EE with 18-month-old mice. Much like what was observed in middle age mice, EE significantly improved GTT when initiated at 18-month of age (Figure 13A). A total of 4 cohorts were maintained in EE till death and combined for lifespan analysis. EE showed an increasing trend of means of lifespan (Figure 13B, C).

## DISCUSSION

The evolutionary theory of aging states that the mechanisms beneficial to coping with environmental demands and resistance to disease are beneficial to lifespan. Moreover, these mechanisms are conserved across species [56]. There is little doubt that the brain plays a commanding role in these lifespan-determining pathways. However, how these neuronal pathways convey signals to the periphery to improve the healthspan of many different organ systems is poorly understood. We propose that the newly defined HSA axis may provide one mechanistic explanation. BDNF, the upstream key component highly responsive to environmental stimuli, could control the HSA axis activity and thereby regulate the phenotype and function of adipose tissue. Adipose tissue, as a principal responsive organ in the periphery of this regulation network, is able to subsequently influence multiple organ systems to change body composition, metabolism, insulin sensitivity, hormones and growth factors, immune functions, cancer and ultimately healthspan or lifespan. Our data demonstrated that middle-age female mice were readily stimulated by EE exhibiting a robust activation of the HSA axis. This study and our previous investigation demonstrate that the key EE-induced features are independent of gender and age— including upregulation of hypothalamic BDNF, reduction of adiposity, drop of leptin, improved glycemic control, and remodeling of adipose tissues. Several effects of EE found in young male animals were not reproduced in older female mice such as decreased serum IGF-1, increased serum adiponectin and corticosterone, and enhanced NK cell activity. The modulations of IGF-1 and adiponectin by EE are likely more related to gender since both factors were not significantly changed in either young female wild type or a spontaneous breast cancer mouse model (data not shown, manuscript under review). The immune analyses of this study were limited to splenic lymphocyte proliferation and NK cell cytotoxicity. Our recent studies using young animals have revealed new effects of EE on immune functions including regulation of thymus and T cell development and modulation of immune cells residing in adipose tissues (manuscripts under review). It is particularly intriguing to investigate how EE regulates different types of immune cells residing in the adipose tissues in aged animals since these cells could play different, possibly even opposite, roles in metabolic adaption along aging process [57,58].

Neuroinflammation, particularly in the aging hypothalamus, may contribute to metabolic syndrome [59], which is thought to be mediated through induction of hypothalamic NFκB or variable SOCS3 signaling in microglia. Of note, inflammatory genes, including NFκB and SOCS3, were downregulated in hypothalamus after long-term EE, which could contribute to the EE’s anti-obesity phenotype. Interestingly, these inflammatory mediators in the hypothalamus can also upregulate sympathetic pathways during normal aging in animal models. It will be interesting to investigate whether EE modulates microglia to exert an anti-inflammatory effect in aged brain.

The activation of the HSA axis is a potent model to decrease fat mass with little or no impact on body weight. EE provides a physiological model to clarify controversies in aging research, e.g. whether weight loss is beneficial to lifespan and whether fat loss with no loss of lean mass is required [60]. EE initiated at middle age promoted healthy aging associated with some metabolic adaptations overlapping with CR. However, there are differences. CR requires sustained reduction of food consumption that is difficult to achieve outside the laboratory. EE led to leanness and resistance to DIO with no suppression of food consumption and instead via increasing energy expenditure. In contrast, CR is associated with reduced metabolic rate. Physical exercise, a model with decreased adiposity but increased energy expenditure, has been shown to be beneficial on healthy aging but unable to extend maximum lifespan [61]. EE provides opportunities for physical exercise. However, our data in previous studies have demonstrated that voluntary running is not sufficient to activate the HSA axis and EE has stronger anticancer and anti-obesity effects than running alone even with overall lower physical activity in young animals [10,11]. The new data in this study also showed that voluntary running failed to reproduce several key metabolic effects of EE in aged mice. Thus, EE is a new model to study the relationship between energy expenditure and aging.

The majority of studies on EE and aging investigate behaviors and neurological diseases [62–67]. Our study is the first assessing EE-induced metabolic adaptations in aging. Nevertheless, we examined a battery of anxiety and depression behavior tests and showed that EE initiated at middle age significantly reduced anxiety-like behaviors consistent with previous reports [63,68]. The mechanisms underlying this anxiolytic effect could be multifactorial and require further investigations. EE can modulate the limbic system in animal models with affective disorder that may or may not have accompanying neurological disease [69]. Besides the hippocampus, other limbic structures include prefrontal cortex, nucleus accumbens, ventral striatum, amygdala and hypothalamus [70]. The hypothalamus, an area integrating metabolism, stress, and immune functions, is highly responsive to EE and could also contribute to the anxiolytic effect. In young animals, EE upregulated BDNF expression in the arcuate nucleus as well as ventromedial (VMH) and dorsomedial (DMH) hypothalamus [10]. The DMH is a brain area not only involved in physiological functions such as metabolism and environmental threats, but also is critically involved in behavioral regulation, particularly fear, anxiety and panic-like disorders [71–73]. Obesity has been linked to neuropsychiatric and anxiety disorders including generalized anxiety disorder, panic disorder, post-traumatic stress disorder, emotional reactivity and cognitive dysfunctions [74,75]. Our data demonstrate that EE induces an anti-obesity and anxiolytic phenotype. The DMH could be a target to study whether the anti-obesity and anxiolytic effects are linked. Recently, it was shown that loss of Crh in the PVH resulted in reduced anxiety behaviors [76]. EE also results in a decrease in Crh in the paraventricular (PVH) hypothalamus (Cao et al., 2011), which could account for the decreased anxiety observed in EE housed mice. It is also possible that global improvement of metabolism associated with the HSA axis activation indirectly influences brain functions and behaviors including anxiety.

**Figure 14:**
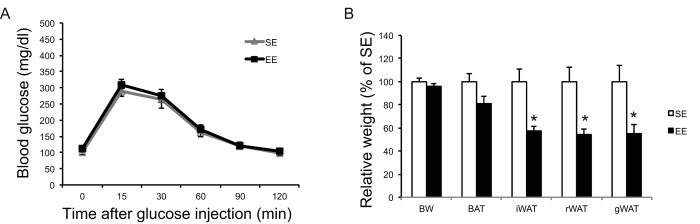
**EE effects on glucose tolerance and adiposity initiated at 2-month of age.** (**A**) Glucose tolerance test after 6-week EE. (**B**) Body and tissue weight at sacrifice after 6-week EE. n=10 per group. * *P*<0.05. Values are means ± SEM.

One new finding of this study is the EE regulation of liver characterized as diminishing aging-related hepatosteatosis, increasing glucose uptake during GTT, and suppressing hepatic glucose production (HGP). These phenotypic changes of liver likely contribute to the enhanced glucose tolerance observed in aging EE mice. Interestingly, short-term EE had no significant impact on GTT in young female mice although adiposity was reduced (Figure 14). It is possible that the aging-related hepatic functional decline allows the observation of the EE modulation of liver. HGP is crucial for systemic glucose homeostasis and is regulated through diverse mechanisms [55]. Impaired suppression of HGP is associated with increased hepatic triglyceride content, a hallmark of non-alcoholic fatty liver disease. And the activation of PKCε serves as one underlying mechanism linking hepatic insulin resistance, hepatosteatosis, and impaired glucose metabolism in metabolic disease models [77]. Our data suggest that the reduced hepatic PKCε level might be a downstream mediator of EE-induced improvement of hepatic and systemic glucose homeostasis. Further assessments of signaling pathways and liver metabolites are underway. Another interesting question is whether the EE’s liver phenotype is regulated via a brain-liver axis, or via the crosstalk between liver and the HSA axis-remodeled adipose tissue. Leptin suppresses HGP [78] whereas lipolysis stimulates HGP [79]. EE resulted in a sharp drop of circulating leptin level and increased lipolysis of adipose tissue. However, HGP was lower in EE mice suggesting the EE-induced adipose remodeling unlikely a key mechanism. Future studies are required to determine whether EE modulates HGP via a brain-liver circuit [80–82].

Lastly, our data showed that mice as old as 18 months were still highly responsive to EE with a significantly improved GTT. Although EE failed to extend maximum lifespan when initiated at the age of 18 months, the means of lifespan of EE mice showed a trend of increase (~ 45 days). Implementation of EE at earlier age may better assess the impact of EE on lifespan.

In summary, EE initiated after middle age led to metabolic adaptations including reduced adiposity, improved systemic glycemic control, increased mitochondrial biogenesis/function, decreased leptin, adipose remodeling, suppressing hepatosteatosis and glucose production. EE also enhanced motor abilities and reduced anxiety. These data suggest that a physically, mentally, and socially active environment can promote healthy aging and a specific brain-fat axis may be one of the underlying mechanisms. Further characterization of the HSA axis and identification of additional mechanisms may reveal potential targets for the prevention and treatment of age-related diseases.

## METHODS

### EE protocol

We housed 10-month old female C57Bl/6 mice (from National Institute on Aging, Aged Rodent Colonies) in large cages (63 cm x 49 cm x 44 cm, 5 mice per cage) supplemented with running wheels, tunnels, igloos, huts, retreats, wood toys, a maze, and nesting material in addition to standard lab chow and water. We housed control mice under standard laboratory conditions (5 mice per cage). All use of animals was approved by, and in accordance with the Ohio State University Institutional Animal Care and Use Committee. Mice were housed in temperature (22-23 ºC) and humidity controlled rooms with food and water *ad libitum*. We fed the mice with normal chow diet (NCD, 11% fat, caloric density 3.4kcal/g, Teklad). In the short-term EE study, mice were sacrificed 6-weeks after EE housing. In the long-term EE study, body weight was monitored weekly until the end of the study at the age of 22 months. Food intake was monitored for 10 weeks as the total food consumption of each cage and represented as the average of food consumption per mouse per day. Rectal temperature was measured at 39-weeks in EE.

### Glucose tolerance test

Mice were injected intraperitoneally with glucose solution (2 g glucose per kg body weight) after an overnight fast. Blood was obtained from the tail at 15, 30, 60, 90, and 120 min after glucose injection. Blood glucose concentrations were measured with a portable glucose meter (Bayer Contour Next).

### Pyruvate tolerance test

Mice were injected intraperitoneally with 15% sodium pyruvate solution (1.5 g sodium pyruvate per kg body weight) after an overnight fast. Blood glucose concentrations were measured with a portable glucose meter as described above.

### Quantitative RT-PCR

We dissected brown and white adipose tissues, amygdala, and hypothalamus and isolated total RNA using RNeasy Lipid Kit plus RNase-free DNase treatment (Qiagen). We generated first-strand cDNA using TaqMan Reverse Transcription Reagent (Applied Biosystems) and carried out quantitative PCR using StepOnePlus Real-Time PCR System (Applied Biosystems) with the Power SYBR Green PCR Master Mix (Applied Biosystems). Primer sequences are available on request. We calibrated data to endogenous control Actb, Ppia, or Hprt1 and quantified the relative gene expression using the 2 ^-ΔΔCT^ method [83].

### Mitochondrial DNA measurement

Total DNA was isolated using the AllPrep DNA/RNA/Protein Mini Kit (Qiagen). Mitochondrial mass was determined by measuring mitochondrial DNA-encoded cytochrome c oxidase subunit I (Cox I) by qPCR. Cox I levels were normalized to Bdnf encoded by nuclear DNA.

### Serum harvest and biomarkers measurement

Trunk blood was collected at euthanasia. We prepared serum by allowing the blood to clot for 30 min on ice followed by centrifugation. Serum was at least diluted 1:5 in serum assay diluent and assayed using the following DuoSet ELISA Development System (R&D Systems): mouse IGF-1, Adiponectin, and Leptin. Glucose and triglycerides were measured using Cayman Chemical colorimetric assay kits. Total cholesterol, triglyceride, and non-esterified fatty acid levels were measured using Wako instruments kits. Glucagon was measured using Glucagon EIA kit (Sigma RAB0202-1KT).

### Hepatic metabolic parameters

Lipid was extracted from liver by chloroform /methanol (2:1 v/v), followed by rinse in 50 mM NaCl and CaCl (0.36M)/Methanol (1:1 v/v) [84]. Hepatic triglycerides quantification was carried out using WAKO instruments kits. Hepatic glycogen content was measured by hydrolysis of liver tissue in acid followed by colorimetric measurement of the resulting glucose [85].

### Adipose tissue immunohistochemistry

We cut paraffin-embeded sections (4 μm) of adipose tissues and subjected the sections to citrate-based antigen retrieval following by incubations with antibodies against UCP1 (Abcam ab10983, 1:1000) or PGC-1α (Abcam ab54481, 1:250). The sections were visualized with DAB and counterstained with hematoxylin.

### Liver histology

Liver was dissected at sacrifice, snap frozen on dry ice, and stored at -80 ºC. For sectioning, liver tissue was embedded in O.C.T. (Sakura Finetek, Torrance, CA) before being sectioned into 15 µm slices on a Leica cryostat. Lipids in frozen liver sections were then stained with an Oil Red O solution (Sigma, St. Louis, MO).

### Western blot

Liver tissues were homogenized in ice-cold Pierce RIPA buffer containing 1x Roche Phosstop and Calbiochem protease inhibitor cocktail III. Blots were incubated overnight at 4 °C with the following primary antibodies: Glucose-6-phosphatase (G6PC, Abcam, #ab83690, 1:500); Phosphoenolpyruvate carboxykinase 1 (PCK1, Cell signaling, #12940, 1:500); Protein kinase C ε (PKCε, Cell signaling, #2683, 1:1000); mature form SREBP1 (Novus Biologicals #NB100-60545, 1:1000); Actin (Cell Signaling #4970, 1:3000). Blots were rinsed and incubated with HRP-conjugate secondary antibody (BIO-RAD). Chemiluminescence signal was detected and visualized by LI-COR Odyssey Fc imaging system (LI-COR Biotechnology, Lincoln, NE). Quantification analysis was carried out with image studio software version 5.2 (LI-COR Biotechnology).

### Body temperature

Rectal temperature was measured at 2 PM for all mice after 5 minutes of sedation with 2.5% isoflurane. The Physitemp BAT -12 rectal thermometer (Clifton, NJ) remained in place for 30 seconds to allow temperature to stabilize before being recorded. Mice were then returned to their home cages to recover.

### Voluntary running

We housed female 10-month-old C57BL/6 mice in rat cages (37 cm x 25 cm x 23 cm) to accommodate two small metal running wheels (11.4 cm diameter, Kaytee, Chilton, WI), 4 mice per cage. Sedentary control mice were housed in standard cages without running wheel.

### Splenocyte proliferation assay

We harvested splenocytes from mice of the 12-month EE at sacrifice. Single-cell splenocyte suspensions were prepared by teasing spleens and passing through 40 μm Cell Strainer. Erythrocytes were depleted with Red Blood Cell Lysis Buffer (Sigma). Splenocytes were washed 3 times and the viability was assessed by Trypan blue exclusion (usually >90%). Splenocytes were seeded in 96-well plates in complete medium (RPMI1640, 25mM HEPES, 2mM L-glutamine, 50μM β-mercaptoethanol, 2g/L sodium bicarbonate, 5% FBS). Quardruplicate of cells from each mouse spleen were stimulated with 0 μg/ml of mitogen or 5 μg/ml Concanavalin-A (Sigma) and cultured for 48-72 hrs. Cell proliferation was determined using the CellTiter 96AQ_ueous_ One Solution Cell Proliferation Assay (Promega). Data were expressed as Stimulation Index = mean OD of wells with Concanavalin-A stimulation/mean OD of the wells without stimulation.

### Cell-mediated cytotoxicity assay

We assayed immune cell cytotoxicity using the CytoTox96 Assay (Promega) according to the manufacturer’s instruction. For NK cell activity, splenocytes were prepared from mice that undergone 12-month EE. Splenocytes were incubated with B16 melanoma cells at various effector: target ratios, the effector NK cells lysed the target cells and LDH release was measured. Each reaction was performed in quadruplicate. The data were calculated using the following formula: % cytotoxicity = (Experimental release- Effector spontaneous release-Target spontaneous release)/(Target maximum release-Target spontaneous release) x 100.

### *In vivo* glucose uptake

The *in vivo* glucose update during a glucose tolerance test was measured using glucose analog tracer 2-[^3^H] deoxyglucose (2-DG) in mice 3-months post initiation of EE following published methods [86,87]. Briefly 2-DG (PerkinElmer, MA) was mixed with regular D-glucose (10µCi/mouse) and injected intraperitoneally. 10-15 µl of blood was collected for glucose specific activity calculation at 0, 15, 30, 60, 90 and 120 min meanwhile blood glucose level was measured with glucometer at each time point of GTT. Then mice were euthanized and liver, adipose tissue and muscle were collected and snap-frozen for further analysis. Adipose tissues, liver, and skeletal muscle samples were homogenized in deironed water, and the proteins were precipitated with 7% ice-cold perchloric acid, and then neutralized by 2.2 M potassium bicarbonate. The aliquot of supernatant was passed through an anion exchange column (Ag-1 x8, Bio-Rad) to trap 2-DG-P. The column was eluted, 2-DG-P was collected, and counted for [^3^H] –radioactivity (LS-6500, Beckman Counter). For blood sample, 2 µl of serum was deproteinized in 3.5% ice-cold perchloride acid (200µl), then neutralized with 2.2 M potassium bicarbonate. The supernatant was counted for [^3^H] –radioactivity. Glucose specific activity (GSC) is calculated by dividing sample radioactivity by glucose concentration. Tissue glucose uptake is calculated by dividing [^3^H]-radioactivity in 2-DG-6-P by mean specific activity of glucose during GTT (120 min) and presented as mmol per mg protein per minute.

### Open field test (OF)

To assess exploration and general motor activity, mice were placed individually into the center of an open square arena (60 cm x 60 cm, enclosed by walls of 48 cm). Each mouse was allowed 10 min in the arena, during which time its activity was recorded and analyzed by TopScan (Clever Sys Inc). The specific parameters measured include distances traveled in the periphery and in the center of the arena (36 cm x 36 cm), the total distance traveled, and the time spent in the center of the arena. The total distance traveled provides a measure of exploratory activity while the time and distance ration of arena center exploration provide an indication of anxiety. The arena was cleaned with 30% ethanol between trials to remove any odor cues.

### Novelty suppressed feeding test (NSF)

Mice were fasted overnight with food removed at 1700h. The testing phase was conduced the next day at 1400h. Mice were individually placed into a brightly lit novel open cage (40 cm x 28 cm x 20 cm). A piece of white filter paper (7 cm diameter) was placed in the center of the cage with a single pre-weighed food pellet. The latency to consumption (first bite of the food pellet) was recorded. The cut-off time was 10 min. To assess if there was any difference in consumptive drive, each mouse was placed in a standard cage with the pre-weighed food pellet after its first bite or at cut-off time if it failed to each within 10 min. The amount of food consumed in 5 min was measured.

### Forced swim test (FST)

Mice were placed individually in a transparent cylinder (21 cm diameter, 24 cm height) containing water (25 ± 2 ºC) to a depth of 15 cm for 6 min. At the end of each trial, mice were dried and returned to their home cage on a heating pad. Trials were video-recorded and a blinded experimenter scored the amount of time mice remained immobile as a measure of depressive-like behavior.

### Cold-Induced defecation (CID)

A large container was filled halfway with ice. A novel cage, smaller than the standard ventilated cage, was placed on top of the ice. A mouse was placed into the smaller cage and a lid was placed on top. After 20 min, the mouse was removed and the number of fecal boli was counted. Mice were allowed to recover in a cage partially on a heating pad for 1 h prior to returning to its home cage. All cages used were cleaned with Spor-Klenz between each animal trial.

### Rotarod treadmill test

Mice were placed on an elevated (30 cm), rotating rod (3 cm in diameter and 6 cm long) once it reached 4 rotations per minute (RPM). Rotation acceleration was set to 20 RPM and after 10 s of the mouse being on the rod, acceleration started. The time on the rod and the speed at which the mouse fell off were recorded. The cut-off time was 5 min. Each mouse was subjected to 2 trials and the apparatus was cleaned with 70% ethanol after each test.

### Body composition by EchoMRI

EchoMRI was used to measure body composition of fat, lean, free water, and total water masses in live mice without anesthesia. EchoMRI imaging was performed with EchoMRI Analyzer at Small Animal Imaging Core of The Dorothy M. Davis Heart & Lung Research Institute, Ohio State University.

### Survival study

A total of four cohorts were recruited for the lifespan study. Each cohort consisted 20 female C57BL/6 mice (18 months old, National Institute on Aging, Aged Rodent Colonies). Each cohort of mice was randomized to live in EE or SE until natural death or reaching the criteria for euthanasia based on an independent assessment by a veterinarian according to IACUC approved protocol.

### Statistical analysis

Data are expressed as mean ± SEM. We used Prism Mac version 6.0f software (GraphPad, La Jolla, CA) and SPSS Statistics v24.0.0.0 (IBM, Armonk, NY) to analyze the following: student’s *t* test for body weight or food intake at single time points, adiposity, body temperature, organ weights, serum ELISAs, behavior, and quantitative RT-PCR data. Mixed analysis of variance was performed on time course measurements (body weight, GTT, PTT).
